# Supplementary material for: High Sensitivity Planar Hall Effect Magnetic Field Gradiometer for Measurements in Millimeter Scale Environments
Source: Micromachines (Basel). 2022 Nov 2;13(11):1898. doi: 10.3390/mi13111898 (PMC9694209; doi:10.3390/mi13111898)
Supplement: Supplementary file 1 [file micromachines-13-01898-s001.zip › SI.pdf]

High sensitivity planar Hall effect magnetic field gradiometer for measurements in millimeter scale environments

Hariharan Nhalil, Moty Schultz, Shai Amrusi, Asaf Grosz, and Lior Klein*

Department of Physics, Institute of Nanotechnology and Advanced Materials,

Bar-Ilan University, Ramat-Gan 52900, Israel and

Department of Electrical and Computer Engineering,

Ben-Gurion University of the Negev,

P.O. Box 653, Beer-Sheva 84105, Israel

(Dated: November 2, 2022)

*Electronic address: Lior.Klein@biu.ac.il

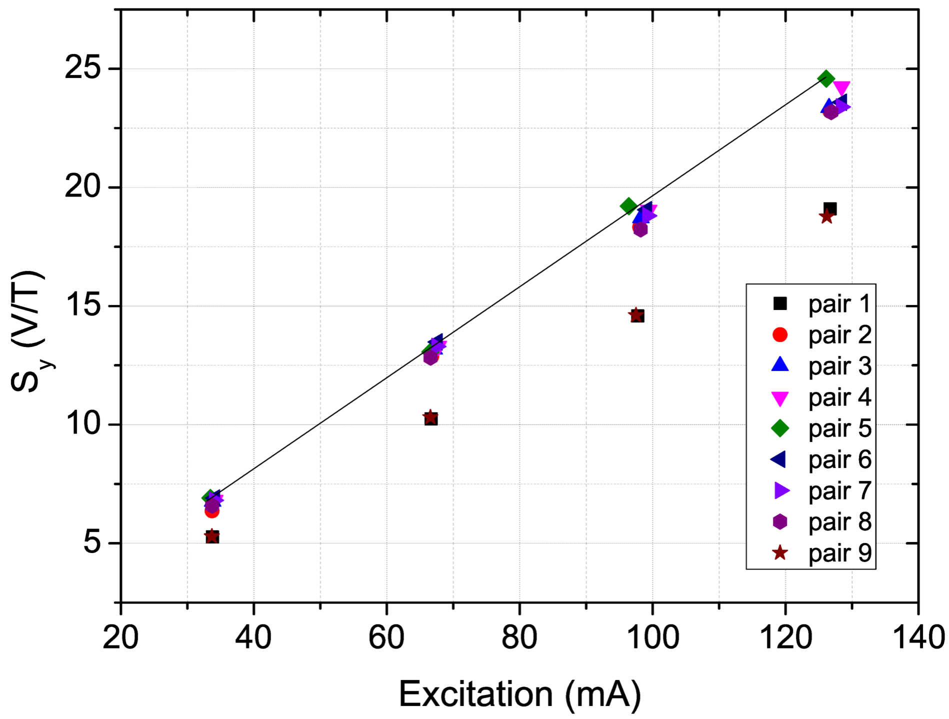

FIG. S1: Excitation current vs Sensitivity (S_y) plot for different excitation currents for all the 9-voltage pairs showing linear dependency. For clarity, only one linear fit is shown (for pair number 9)

TABLE SI: EMNs for 2 different excitation currents for the 9-voltage pairs for 50, 10, 1 and 0.1 Hz frequencies.

V-pair No:	Exc. Curr. (mA)	EMN @ 50 Hz (pT/$\sqrt{Hz}$)	EMN @ 10 Hz (pT/$\sqrt{Hz}$)	EMN @ 1 Hz (pT/$\sqrt{Hz}$)	EMN @ 0.1 Hz (pT/$\sqrt{Hz}$)
1	~ 33	93	111	269	989
	~ 67	68	79	462	1995
2	~ 33	83	92	198	734
	~ 67	44	63	202	807
3	~ 33	76	82	155	574
	~ 67	43	58	172	681
4	~ 33	76	82	151	512
	~ 67	40	57	166	594
5	~ 33	81	90	172	532
	~ 67	50	68	208	823
6	~ 33	76	81	151	551
	~ 67	38	54	155	543
7	~ 33	76	83	158	535
	~ 67	43	58	175	708
8	~ 33	84	92	187	694
	~ 67	46	67	222	915
9	~ 33	91	106	264	1068
	~ 67	66	77	422	1915
