# Supplementary figures and images for: High Sensitivity Planar Hall Effect Magnetic Field Gradiometer for Measurements in Millimeter Scale Environments
Source: Micromachines (Basel). 2022 Nov 2;13(11):1898. doi: 10.3390/mi13111898 (PMC9694209; doi:10.3390/mi13111898)

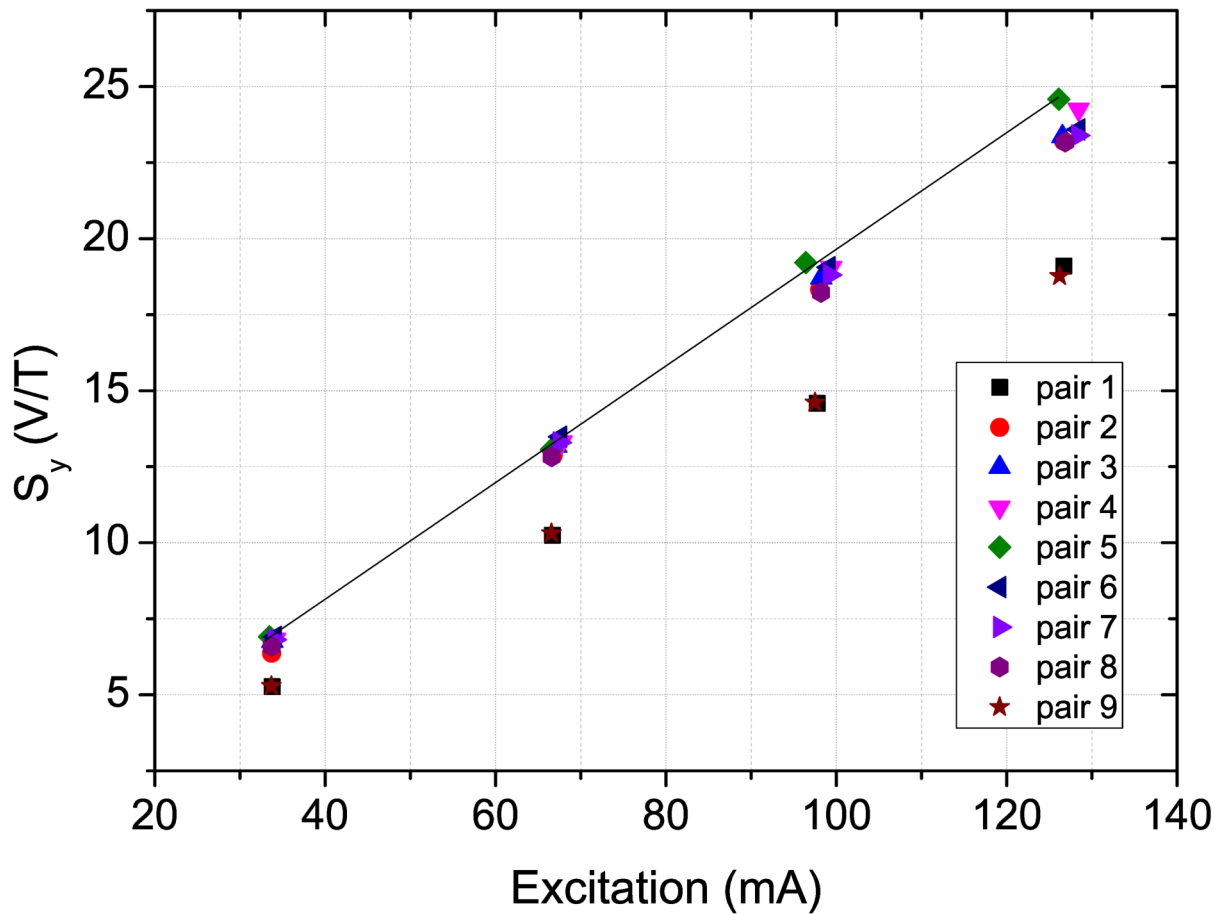

Supplement: Supplementary file 1 [file micromachines-13-01898-s001.zip › FIG_S1-eps-converted-to.pdf]
